# Do intoxicated witnesses produce poor facial composite images?

**DOI:** 10.1007/s00213-018-4989-2

**Published:** 2018-08-17

**Authors:** S. J. Bayless, A. J. Harvey, W. Kneller, C. D. Frowd

**Affiliations:** 1 Department of Psychology, University of Winchester, Sparkford Road, Winchester, SO22 4NR UK; 2 Department of Psychology, University of Portsmouth, Portsmouth, UK; 3 University of Central Lancashire, School of Psychology, Preston, UK

**Keywords:** Alcohol intoxication, Facial composite, Face memory

## Abstract

**Rationale:**

The effect of alcohol intoxication on witness memory and performance has been the subject of research for some time, however, whether intoxication affects facial composite construction has not been investigated.

**Objectives:**

Intoxication was predicted to adversely affect facial composite construction.

**Methods:**

Thirty-two participants were allocated to one of four beverage conditions consisting of factorial combinations of alcohol or placebo at face encoding, and later construction. Participants viewed a video of a target person and constructed a composite of this target the following day. The resulting images were presented as a full face composite, or a part face consisting of either internal or external facial features to a second sample of participants who provided likeness ratings as a measure of facial composite quality.

**Results:**

Intoxication at face encoding had a detrimental impact on the quality of facial composites produced the following day, suggesting that alcohol impaired the encoding of the target faces. The common finding that external compared to internal features are more accurately represented was demonstrated, even following alcohol at encoding. This finding was moderated by alcohol and target face gender such that alcohol at face encoding resulted in reduced likeness of external features for male composite faces only.

**Conclusions:**

Moderate alcohol intoxication impairs the quality of facial composites, adding to existing literature demonstrating little effect of alcohol on line-up studies. The impact of intoxication on face perception mechanisms, and the apparent narrowing of processing to external face areas such as hair, is discussed in the context of alcohol myopia theory.

According to recent UK statistics, around 70% of violent incidents that took place in public places between 2012 and 2014 were alcohol related (ONS 2015). Victims perceived the perpetrator to be intoxicated in around 53% of incidents and reported themselves to have been intoxicated in around 20% of cases. Data from the USA suggest that witnesses are deemed by police officers to be intoxicated in around 41% of violent crimes (Evans et al. 2009). In another survey, 33% of testimonies heard in court were made by witnesses who were intoxicated at the time of the incident (Palmer et al. 2013). It is therefore common to find witnesses and victims to be under the influence of alcohol at the time of being involved in, or witnessing a crime, making it important to establish their ability to accurately report details of the event.

A comprehensive body of research has demonstrated the detrimental effect of alcohol on attentional processes in experimental paradigms (Bayless and Harvey 2017; Canto-Pereira et al. 2007; Harvey 2016; Harvey et al. 2017; Schulte et al. 2001). Such effects extend to visual scene perception (Clifasefi et al. 2006; Harvey et al. 2013a), and the volume of recalled information of visual event details (Hagsand et al. 2013a; Schreiber Compo et al. 2011; van Oorsouw and Merckelbach 2012). Similarly, memory encoding is adversely affected by alcohol (e.g. Sőderlund et al. 2005), and alcohol disrupts effortful but not automatic memory processing (Crossland et al. 2016; Tracy and Bates 1999). On the other hand, memory is facilitated if alcohol is ingested following learning (retrograde facilitation, e.g. Carlyle et al. 2017). The effect of alcohol intoxication on memory is important forensically, as it predicts performance of witnesses intoxicated at the scene of a crime.

Studies evaluating the effect of alcohol on witness memory generally focus either on testimony of events or the ability to identify a suspect, for instance in a line-up. Despite the deleterious effect of alcohol on memory encoding, many studies of eyewitness testimony do not report an adverse effect of alcohol on the accuracy of recalled details, but some report that fewer units of information may be recalled (for reviews see Hagsand et al. 2017, and Hildebrand Karlén 2018). One of the first studies to systematically address the effects of alcohol intoxication on witness memory was carried out by Yuille and Tollestrup (1990). The authors reported that intoxicated participants were able to recall less information than sober counterparts in immediate and delayed recall tests, but that line-up identification of the target was largely unimpaired by alcohol. Several recent studies that have specifically used forensically relevant line-up identification procedures also report no significant impairment in target identification as a result of mild-to-moderate alcohol intoxication (Altman et al. 2018; Colloff and Flowe 2016; Flowe et al. 2017; Hagsand et al. 2013b; Harvey et al. 2013b; Kneller and Harvey 2016). In all but one of these studies, participants were intoxicated at the time of witnessing a staged event or video of a crime, and performed the line-up identification in a sober condition up to a week later. The exception is the recent study by Altman et al. (2018) whose alcohol participants remained intoxicated throughout the encoding and retrieval phase of the line-up task. The only report of a negative effect of alcohol on identification accuracy is that of Dysart et al. (2002), who found a positive relationship between the intoxication level of observers and the likelihood of them falsely identifying a foil. However, these authors employed a “showup” identification procedure, requiring a forced-choice decision about the target’s identity based on a single photograph of the target or a similar looking foil. The suggestive nature of this protocol may increase the rate of false identifications in target absent trials relative to line-up procedures (Steblay et al. 2003).

While findings from line-up studies suggest that intoxication at the time of witnessing (i.e. encoding) a target face does not impair subsequent identification performance, two standard old-new face recognition studies have shown alcohol impairments (Harvey 2014; Hilliar et al. 2010). In an investigation of the effect of alcohol intoxication on the own-race (unfamiliar) face recognition bias (ORB), Hilliar et al. (2010) reported higher false identification rates and lower hit rates for intoxicated participants relative to sober controls, particularly for own-race faces. Following Dysart et al. (2002), the authors argue that intoxication restricts face encoding resulting in lower identification accuracy.

While Hilliar et al. do not propose a specific mechanism for their reported “lazy” alcohol encoding effect, they do explain their findings with reference to Dysart et al. (2002). Dysart et al. (2002) suggest that their intoxicated witnesses were more likely to falsely identify the foil because of a narrowing of attention on to a particularly salient facial feature, which in their study was possibly the distinctive hairstyle the foil shared with the target female. Dysart et al. (2002) explained their findings with respect to alcohol myopia theory (AMT, Steele and Josephs 1990). AMT proposes that alcohol intoxication reduces attentional capacity and, as a consequence, limited processing resources are allocated to stimuli that are immediately available or salient. Several other studies of alcohol effects on attention and memory have discussed their findings in the context of AMT (e.g. Hagsand et al. 2013a; Schreiber Compo et al. 2011). Stimuli are typically classified as high/low salience depending on the nature of the stimulus and its spatial location (central or peripheral to a scenario). AMT can reconcile the null findings from most line-up studies firstly because faces tend to be highly salient stimuli in our environment (Little et al. 2011) and secondly, in the context of a mock crime, the perpetrator’s face is central to the scenario. When the face is the only or key stimulus, however, it is more difficult to predict alcohol’s effect according to AMT, as it is not straightforward which area of a face is considered salient or central in a given context. At the time of writing this manuscript, the authors are aware of only two studies which have tested alcohol effects in a face memory task with respect to AMT (Colloff and Flowe 2016; Harvey 2014). Colloff and Flowe (2016) found no support for AMT in their study which tested the effect of alcohol and salient facial characteristics on face memory. Harvey (2014) reported that alcohol narrowed the range of eye fixations participants made towards the central nose region of each stimulus face, rather than on to the external hair region, while observing no adverse effect of alcohol intoxication on face recognition performance. This visual narrowing was explained as an effect of AMT, but as it did not impact on face memory, this does not provide strong support for AMT to predict the effects of alcohol on face memory processes.

A forensically important method for identification that has not been studied in the context of intoxicated witnesses is the use of facial composite images. Line-up identification procedures rely on investigators having information about potential suspects, for instance from closed-circuit television (CCTV) footage. However, in the absence of such information, investigators rely on facial composite construction techniques to create a visual likeness of an offender, and these images are then used to identify the suspect (Frowd et al. 2014). As mentioned above, it is not uncommon for witnesses of a crime to be intoxicated at the time of the event yet, to date, no published study to our knowledge has examined the effect of witness intoxication on facial composite construction.

Facial composites have been used as an important forensic method of identifying potential perpetrators of crime for about 40 years (see Frowd et al. 2012, 2014). Typically, systems rely on a witness to recall the appearance of individual features of the face from memory. Then, with the help of a forensic artist or police practitioner, the individual’s facial elements (nose, ears, eyes, mouth, etc.) are either sketched or selected from a database using a specialised software tool to create the face. Research has demonstrated that recalling an unfamiliar face from memory, in order to assemble facial elements, is inferior to recognising such a face when presented with photographic face stimuli (see Frowd et al. 2012, for discussion). Alternatives to the feature-based composite construction procedures use a whole face (holistic) approach. This relatively new method creates a face composite by “breeding” together several faces selected by the witness based on similarity to the remembered target face. One such system is EvoFIT (Frowd et al. 2008) which has been used successfully in laboratory research and with witnesses and victims of crime (Frowd et al. 2012) demonstrating a greatly improved identification rate compared to traditional systems (e.g. EFIT, Solomon et al. 2012). The quality of the facial composites provided by witness participants is usually evaluated either by a naming procedure (Frowd et al. 2012) or by recording likeness ratings of the facial composite compared to the target face that it is intended to resemble (Frowd et al. 2008). In previous research evaluating composite images, the external face features (e.g. hair) of unfamiliar targets were typically remembered and constructed more accurately by witness participants than internal facial features (e.g. eyes, nose, mouth) using sorting and naming procedures respectively (Frowd et al. 2008, 2012); the effect has, to date, not been demonstrated using likeness ratings. The external features’ advantage in those studies is consistent with research demonstrating that external features are an important factor in unfamiliar face perception (e.g. Bruce et al. 1999; Hancock et al. 2000). Longmore et al. (2015) account for the importance of external features for recognising unfamiliar faces by referring to work by Bruce (1994) and Burton (2013; cited in Longmore et al. 2015), which highlights the difficulty of recognising the same face in different views or perspectives. They argue that external features, especially hair, provide more stable and common perceptual information than internal features which are more susceptible to variation according to facial expression and the observer’s viewpoint. Familiar faces, on the other hand, are better recognised by their internal than external features (e.g. Ellis et al. 1979) and this focus on internal features develops as exposure to the face increases (Bonner et al. 2003; Clutterbuck and Johnston 2005; Osborne and Stevenage 2008). However this external features’ advantage is culture specific such that an internal-feature advantage for unfamiliar faces is reported for participants from cultures where hair is covered (Megreya and Bindemann 2009, 2017).

To date, research investigating the ability of intoxicated witnesses to identify potential suspects has focused on line-up procedures. While there is growing evidence to suggest that moderate levels of intoxication do not impair the accuracy of identifying a suspect from a line-up (e.g. Hagsand et al. 2013b; Harvey et al. 2013b; Kneller and Harvey 2016), it is important to evaluate whether this level of reliability extends to witnesses who provide a facial composite of an offender. The literature addressing the effect of alcohol intoxication on basic memory processes such as word list learning has indicated adverse effects on the volume of recalled information, especially for tasks requiring more effortful processing (Tracy and Bates 1999) or where there is less opportunity for practice (Garfinkel et al. 2006). Furthermore, several studies have demonstrated a more profound effect of intoxication on memory recall than on recognition for various tasks, including the retrieval of event details (Crossland et al. 2016), word lists (Duka et al. 2001), and pictorial information (Söderlund et al. 2005). It is therefore plausible that construction of facial composites is differently affected by alcohol than recognition in line-up procedures.

In the present study, we investigated the potential effect of alcohol intoxication on the quality of facial composite images constructed by participants intoxicated or sober at the time of encoding the face, and either intoxicated or sober at the time of facial composite construction, 24 h later. The resulting facial composite images were evaluated by an independent group of participants, who compared each composite image with a photograph of the target person that the composite image was intended to represent. We used a factorial design for intoxication at encoding and retrieval of information for two reasons. Firstly, there is evidence that witnesses can be intoxicated at the time of giving evidence, even if this is days after witnessing the event (Evans et al. 2009). Secondly, only a few studies have investigated the effects of intoxication at the time of retrieving information (see Schreiber Compo et al. 2017).

Previous research with Caucasian faces and Caucasian participants has consistently demonstrated a processing advantage for external facial features when learning unfamiliar faces. In order to examine the impact of alcohol intoxication on this effect, the present composite stimuli were presented to evaluator participants in three conditions: as full facial images, as internal face features (digital editing of the image to mask the ears, hair and neck), or as external features (the inverse of the internal images). Given the importance of encoding hair for unfamiliar face discrimination (Frowd et al. 2012; Longmore et al. 2015), we expected the external region of our face composites to receive the highest likeness ratings overall. If alcohol restricts fixations towards the internal facial region during face encoding, as suggested by Harvey (2014), this should be reflected in lower likeness ratings for the external region of composites produced by participants intoxicated while encoding the target face. Alternatively, if alcohol narrows attention on to salient external face regions during encoding, such as hair (e.g. Dysart et al. 2002), then we expected no effect of alcohol intoxication on the quality of external face composite features.

It is also the case that the external features of faces typically differ between men and women due to characteristic hairstyles (a greater range of lengths and styling for women, which was reflected in the target faces in our study) and there is evidence to suggest a gender effect for composite naming showing an advantage for female composite faces (Frowd et al. 2012). Therefore, we expected target face gender to interact with alcohol effects at the time of encoding the faces, such that alcohol at encoding leads to an advantage for external face features when these are more salient (in female target faces, in line with Dysart et al. 2002). Although our predictions relate to the salience of external face features, we are not explicitly framing our predictions according to AMT, as it is not possible to provide a generalised prediction regarding the salience of different face regions. However, since the recall element of composite construction is cognitively demanding, and alcohol depletes attentional resources (Steele and Josephs 1990), we also predicted that composites produced by intoxicated participants would receive lower likeness ratings than those constructed by sober participants. We therefore anticipated that the largest reduction to likeness ratings would be for composites produced by participants intoxicated during both encoding and retrieval of the target face.^1^

## Method

### Participants

#### Composite construction

The composite images were constructed by a total of 37 undergraduate students who participated in exchange for course credit or a £20 honorarium. Data from 5 participants were excluded because of technical errors related to saving the composite images, or ineligibility to take part in one of the alcohol conditions (in which case the participant was assigned to known placebo, rather than prevented from participating, and their composite images were not used). The final sample consisted of 17 females and 15 males with ages ranging from 18 to 28 (*M*_age_ = 20.62 years, *SD*_age_ = 2.38 years).

The experiment was advertised as a study of the effects of alcohol intoxication on memory and face processing. Individuals who expressed an interest were provided with an information sheet and an alcohol screening form to rule out any contraindications to taking part in the study. The screen was designed to ensure that participants were a minimum of 18 years of age (legal UK drinking age), had no medical concerns that preclude drinking of alcohol, were within the weight range (55–95 kg) and were regular social drinkers who had consumed at least six units of alcohol in one sitting within the previous 3 months. All aspects of the study and study materials were considered and approved by the University’s research ethics committee and the study followed the guidelines detailed in the British Psychological Society’s Code of Ethics and Conduct (2009).

#### Composite evaluation

The composite images were rated by a second group of participants who had not been involved in composite construction. A total of 52 participants took part (8 male, 44 female, ranging in age from 18 to 53 (*M*_age_ = 21.57, *SD*_age_ = 6.55 years). All were undergraduate students in Psychology. The materials and procedure were approved by the University’s research ethics committee.

### Design

Thirty-two face composite images were constructed that were produced by participants randomly allocated to one of four conditions derived from crossing the factor “alcohol” (defined below) and the two composite construction phases: placebo both at encoding and at construction; placebo at encoding and alcohol at construction; alcohol at encoding and placebo at construction; and alcohol both at encoding and at construction. The likeness ratings were provided by a different group of participants who had not taken part in composite construction, and were not involved in the alcohol manipulation. Participants providing the likeness ratings were randomly allocated to one of three facial composite image conditions: full composite face, internal features only and external features only. The study consisted of a 2 (treatment at encoding: alcohol vs. placebo) x 2 (treatment at composite construction: alcohol vs. placebo) x 2 (target face gender) x 3 (composite stimulus condition: full face, internal features, external features) mixed design with composite stimulus as the only between subjects’ variable. The dependent variable was likeness rating of the composite image compared to the original photograph of the individual, measured using a Likert scale ranging from 1 (very poor likeness) to 7 (excellent likeness).

### Materials and measures

#### Composite construction

##### Breath alcohol measurement

The breath alcohol measurement (BrAC) in participants’ deep lung air was recorded using a Draeger Alcotest breathalyser, which is approved for use by the UK Home Office and had been serviced and calibrated according to the manufacturer’s recommendation. The unit of measurement was grams of alcohol per 210 L of breath (g/210 L). This measurement is equivalent to grams of ethanol per 100 ml of blood (blood alcohol concentration, BAC). The drink driving limit in England and the USA is 0.08% BAC which is approximately BrAC of 0.08 g/210 L of breath. For ease of reading, all BrAC measurements will be expressed as percentages (where BrAC% refers to g/210 L of breath).

##### Stimulus videos

Stimuli consisted of colour videos, showing the head and shoulders of 8 target identities, 4 female and 4 male, facing the camera and describing their hobbies and interests. The individuals were members of faculty at one of the author’s former academic departments, which is overseas, thus eliminating the likelihood that the target faces would be familiar to any of our participants. Each video recording lasted for 1 min.

##### EvoFIT

Composite face images were constructed using EvoFIT (Frowd et al. 2014) software. EvoFIT is a computerised face composite construction system that uses a holistic approach to construction by “breeding” the face models selected by witnesses. EvoFIT relies on participants choosing overall similar looking faces to the target, rather than recalling specific target face features. Participants were guided through this selection process for two complete iterations, after which they were given the opportunity to enhance the appearance of the face by adjusting a range of facial features including skin tone, texture and fullness of the face, and finally add an appropriate hair style. For further procedural details, see Fodarella et al. (2015).

#### Composite evaluation

**Fig. 1:**
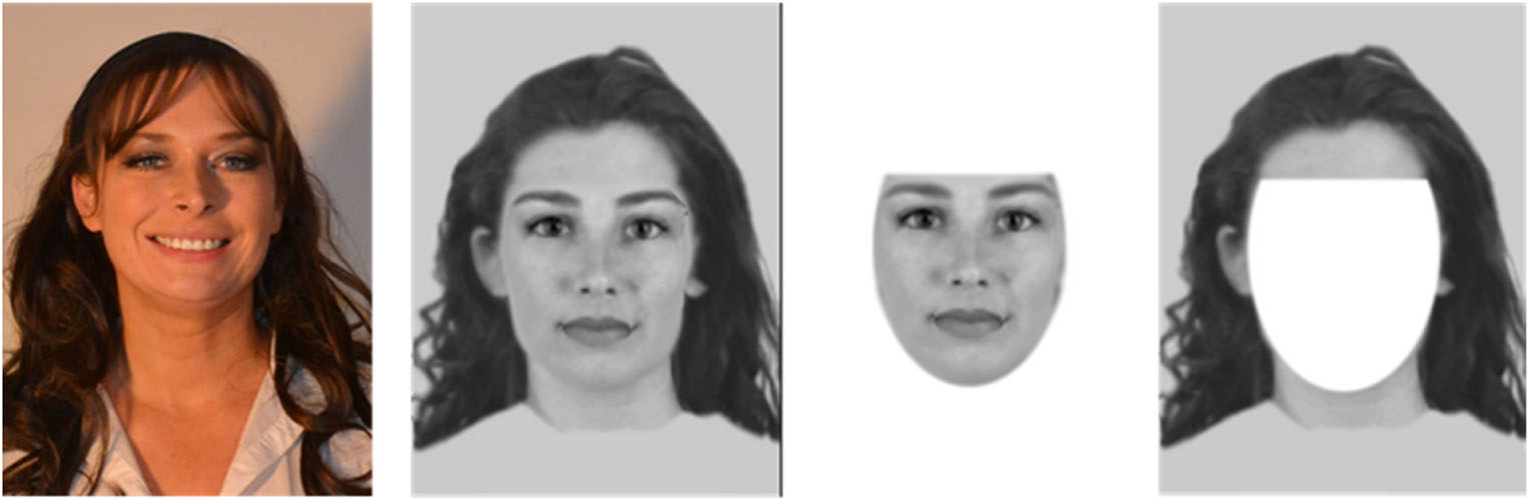
Examples of target photograph, an example full face composite and the stimuli created for internal and external face parts (from left to right respectively)

For each target, a still image was obtained from the video used in the face encoding stage. The image was cropped to represent a passport-style photo presented in colour with the head and shoulders visible. The composite images were greyscale and cropped to similar dimensions as the target image. Composites were presented as a full face, the internal features or external features only. For the part face conditions, the image was manipulated using Adobe Photoshop software, with the internal features cropped out of the image using the elliptical and rectangle selection tool, and the edges smoothed using the blur tool. The external features image was the inverse selection to the internal features stimulus (see Fig. 1 for an example). Images were presented on A4 paper, orientation landscape, with the target image presented on the left and the composite image on the right. Image size was 7 × 9 cm.

### Procedure

#### Composite construction

The experiment was advertised as a study investigating the effects of alcohol intoxication on memory for faces. Participants were asked to complete a screening form before attending the lab (see information in the “Participants” section). Any individuals who did not satisfy the initial screening criteria were thanked for their interest but not invited to participate. Participants were asked to eat a light meal 1–2 h before attending the study in order to attempt to match the rate of alcohol absorption. Participants who did pass initial screening but were found to not meet the criteria fully on the day of the experiment were assigned to a known control condition and their data was not used. On the first day of the study, participants were provided with information about the study and were invited to ask questions. During briefing and consent, the participant was asked to complete a waiver confirming their awareness that they should not drive or take part in any risky activity should they leave the research venue with a breath alcohol concentration at or above 0.08% at the end of the study. Nevertheless, all participants were advised to remain in the laboratory until their intoxication level had dropped below the legal driving limit.

Participants were randomly assigned to one of four conditions, but were naïve to the allocated treatment: (1) placebo at encoding, placebo at construction; (2) alcohol at encoding, placebo at construction; (3) placebo at encoding, alcohol at construction; and (4) alcohol at encoding, alcohol at construction. The study employed a placebo design in line with other recent research (Clifasefi et al. 2006; Harvey 2016; Harvey et al. 2013a, b; Schreiber Compo et al. 2011) in an attempt to control for alcohol expectancy effects. It has been demonstrated that alcohol expectancy can result in participants adjusting their effort or behaviour in order to compensate for their anticipated lower performance under the influence of alcohol (Fillmore and Blackburn 2002; Testa et al. 2006). Testa et al. (2006) have suggested the use of placebo conditions to address these possible effects. All eight target face identities were represented in each of the four experimental conditions. The order of target alcohol condition combinations was then randomised resulting in 32 unique combinations of target face and alcohol condition at encoding and construction phases. Participants were randomly allocated to one of these combinations.

The alcohol administration schedule used in this study is consistent with recent studies using a comparable procedure to achieve the desired BrAC levels at the start of the experimental tasks (Clifasefi et al. 2006; Harvey 2016; Harvey et al. 2013a, b; Schreiber Compo et al. 2011). Before commencing the study, a BrAC reading was taken to ensure a baseline of 0%, and the participant’s weight recorded to the nearest kilogram. Participants were then invited to sit in an adjoining room while their drink was being prepared. For participants in the alcohol-at-encoding conditions (conditions 2 and 4), the drink consisted of 2 ml of vodka (37.5% ABV) per kilogram of bodyweight (adjusted to 1.8 ml for female participants; Mumenthaler et al. 1999), which was topped up to 450 ml of drink with orange juice, and mixed thoroughly. Participants in the placebo-at-encoding conditions (conditions 1 and 3) received 450 ml of orange juice, with 2 ml of vodka dispersed on the surface of the drink, and the outside of the glass misted (three sprays) with vodka to create the smell of an alcoholic beverage. Participants were asked to consume their drink gradually over the course of 15 min, followed by 15 min rest before task commencement. Participants were offered magazines to read during this time.

Following the 30-min interval, a second BrAC reading was taken (reading not disclosed to participants) and participants were asked for their subjective level of intoxication on a scale from 1 (“I feel completely sober”) to 10 (“I feel extremely drunk”). The researcher seated the participants at a laptop equipped with headphones and instructed the participant to play the cued video file once the researcher had left the room. Videos were stored in numbered folders to avoid the researchers seeing the face stimuli. The researchers who carried out the data collection were blind to the identities of the target faces until after the data collection for this study was complete, to avoid any unintentional bias during the facial composite construction phase. Following video presentation, the participants completed a further task which lasted 5 min, unrelated to this study. A third BrAC reading was taken, and if at or above the legal driving limit (0.08%), participants were encouraged to wait in the laboratory for their BrAC to return to a lower level. Participants who had earlier signed the waiver to leave despite still being intoxicated at or above the 0.08% BrAC level were reminded not to drive or engage in any other activity that could be dangerous for the rest of the day.

Participants attended a second test session 24 h later, following the same preparatory instructions as previously. The consent and waiver procedure was identical to the previous day, as was the administration of the alcohol and placebo drinks. In total, three BrAC readings were taken: the first to confirm sobriety on arrival, the second 30 min after the start of beverage consumption, and the final reading after the completion of the facial composite.

The face composite construction procedure began with a structured verbal recall of the appearance of the face seen in the video the previous day. Participants were asked to think back to the person they saw in the video the previous day, and once they had a good mental image of the target’s face, to describe anything they could recall about it. The researcher made written notes of general descriptions. If the participant did not mention gender, age and ethnicity, the researcher prompted for this information, but no other specific questions were probed. Based on target gender, ethnicity and age as described by the participant, the researcher selected the appropriate face database in EvoFIT. If the target face was recalled as wearing glasses (two male and two female models wore glasses in the video), this option was selected by the researcher, and the participant indicated appropriate frames from a selection of shapes and sizes available on the database. The participant was then presented with 18 greyscale faces (with no external features such as hair, but if selected, glasses were presented), and asked to indicate two that had the best likeness to their mental image of the target in terms of face shape. Participants pointed at their selections and the researcher selected these by mouse-click. This selection process was repeated for two more sets of 18 faces, after which the participant was asked to identify and indicate the two faces with the best likeness in terms of face shape from the six that had been pre-selected in the previous 3 rounds. Based on these selections, another set of 18 faces were presented, and the participant carried out the same selection process but based on the texture and colouring of the face. After selection of a best likeness at this stage, this whole process was repeated a second time, before a final face was presented. At this point, the participant was invited to make adjustments to a series of internal features of the face, if they wished. This was done by the researcher using a slider, for example to change the tone of the skin, narrow or widen the face or alter qualitative characteristics such as the trustworthiness and friendliness. The researcher presented each option in the neutral state (central position of slider) and then demonstrated the transitions to both extreme ends of the adjustment scale. The participant was then asked to verbally instruct the researcher to adjust the settings (e.g. “move the slider slightly to the left”). There was a final opportunity for the participant to review the face composite at this stage. If there was nothing else the participant wished to change, the external features were selected. The participant was asked to indicate appropriate hair styles from the database and these could be manually adjusted as required using an analogous process as for the adjustment of internal features. The final face composite was completed on average within 30 min. At this point, a third BrAC reading was taken. This was disclosed to participants and they were invited to stay in the lab if their BrAC was not below 0.08% (this only applied to two participants). Participants were debriefed and offered £20 compensation or course credit as appropriate.

#### Composite evaluation

Participants were recruited through the departmental research participation scheme, and offered course credit. Participants were randomly allocated to a stimulus condition (full face, internal or external features), provided with a brief background about the study, and the rationale for the likeness ratings was outlined. The rating scale consisted of a Likert scale ranging from 1 (very poor likeness) to 7 (excellent likeness), was presented visually and remained visible throughout the ratings. Stimuli were presented in a different random order for each person, on A4 paper, with the target image on the left and the composite image on the right. Participants gave a verbal likeness rating for each composite in their own time, after which the next image was presented. A total of the 32 images were presented to each participant. The task took no more than 10 min, whereupon participants were debriefed and thanked for their time.

## Results

### Breath alcohol concentrations

Breath alcohol concentration was measured at three time points during the experiment: BrAC_1_ was on arrival to ensure the participants were sober, (*M* = 0.00, *SD* = 0.00); BrAC_2_ was taken 30 min after the start of consuming the drink; and BrAC_3_ at the end of the experimental session. On day 1, this was approximately 30 min after BrAC_2_, on day 2 between 30 and 45 min after BrAC_2_. For participants receiving alcohol, BrAC_2_ ranged from 0.03 to 0.09 (*M* = 0.06, *SD* = 0.02) on day 1 and from 0.02 to 0.09 (*M* = 0.05, *SD* = 0.02) on day 2. BrAC_3_ ranged from 0.03 to 0.09 (*M* = 0.05 *SD* = 0.01) on day 1 and 0.03 to 0.09 (*M* = 0.05, *SD* = 0.02) on day 2. Mean intoxication across both days and time points was BrAC 0.05% (*SD* = 0.018). On average participants in this study therefore reached a moderate level of intoxication, below the drink driving limit. Mean subjective intoxications following a placebo drink were lower (*M* = 1.03, *SD* = 1.43), than when following an alcoholic drink (*M* = 4.25, *SD* = 2.01), and this difference was statistically significant, *t*(62) = 7.38, *p* < .001, *d*′ = 1.36. The correlation between subjective intoxication at the time of BrAC2 and the actual intoxication level did not reach statistical significance, *r*(62) = .34, *p* = .06. We tested for potential alcohol priming effects, whereby the alcohol condition on the first day may influence the expectation and subjective experience the following day. The subjective intoxication ratings for day 2 were categorised according to condition (placebo or alcohol) on day 1, and the day 2 ratings for the two experimental groups compared using a repeated measures ANOVA. Although there was a main effect of condition as expected, *F*(3, 28) = 5.93, *p* = .003, *η*_*p*_^2^ = .39, the critical paired comparisons were not significant. Ratings from those receiving placebo on day 2 following alcohol on day 1 (*M* = 0.50) were no different than ratings for those receiving placebo on day 2 following placebo on day 1 (*M* = 1.63), *p* = .177. The same pattern held for those receiving alcohol on day 2 following alcohol on day 1 (*M* = 3.88) vs. alcohol on day 2 following placebo on day 1 (*M* = 3.88), *p* = 1.00.

### Composite evaluation

The quality of the composite images was measured by likeness ratings obtained from a group of participants who had not been involved in the construction phase of the study (see Frowd et al. 2007). Participants provided ratings across the three composite stimulus conditions: full composite faces stimuli (*N* = 17), stimuli consisting of only internal features (*N* = 17) and stimuli consisting only of external features (*N* = 18). Ratings were obtained for each of the 32 facial composite images and then the group mean for each treatment group was calculated and shown in Table 1.

**Table 1:** Mean likeness ratings for factorial combinations of alcohol condition at encoding and construction, by male and female target faces and composite construction condition. Values in parentheses are one standard error of the mean. *P*_enc_, placebo at encoding; *P*_con_, placebo at construction; *A*_enc_, alcohol at encoding; *A*_con_, alcohol at construction

	Full face (*n* = 17)	Internal features (*n* = 17)	External features (*n* = 18)
Combination of alcohol conditions at encoding and construction			
Male target faces			
*P*_enc_ *P*_con_	3.06 (0.20)	3.06 (0.20)	4.18 (0.20)
*P*_enc_ *A*_con_	3.54 (0.21)	3.69 (0.21)	4.43 (0.20)
*A*_enc_ *P*_con_	3.59 (0.18)	3.74 (0.18)	3.50 (0.18)
*A*_enc_ *A*_con_	3.19 (0.18)	3.97 (0.18)	3.18 (0.18)
Female target faces			
*P*_enc_ *P*_con_	3.50 (0.20)	3.06 (0.20)	3.79 (0.20)
*P*_enc_ *A*_con_	2.96 (0.18)	2.62 (0.18)	4.10 (0.18)
*A*_enc_ *P*_con_	2.90 (0.23)	2.63 (0.23)	4.15 (0.23)
*A*_enc_ *A*_con_	3.13 (0.21)	2.81 (0.21)	3.47 (0.20)

The mean likeness ratings were analysed using a 2 (encoding state) x 2 (construction state) x 3 (composite condition) x 2 (target face gender) mixed-design ANOVA. The factors encoding state and construction state refer to the alcohol treatment condition (alcohol or placebo) assigned to the participant at the two phases of the composite construction process. The variable composite condition refers to the composite stimulus that was being rated (full face, internal or external features), and target face gender indicates whether the face stimuli was male or female. All analyses were two-tailed.

The results of the ANOVA are summarised in Table 2. The first prediction was that regardless of alcohol treatment, the external facial features of composite images would receive higher ratings than internal or full face composites. This prediction was supported, as the main effect of composite condition was significant, *F*(2, 49) = 8.27, *p* = .001, *η*_*p*_^2^ = .25, and a post hoc Tukey HSD test indicated an advantage for external face parts (*M* = 3.85, *SE* = 0.13) over both full face composites (*M* = 3.23, *SE* = 0.13, *p* = .004 95% CI [0.18, 1.06]) and internal face parts (*M* = 3.20, *SE* = 0.13, *p* = .002, 95% CI [0.22, 1.09]). Contrary to our prediction, there was no significant effect of alcohol treatment at the time of composite construction on overall likeness ratings, *F*(1, 49) = 0.01, *p* = .93, *η*_*p*_^2^ < .001; however, there was a significant main effect of alcohol treatment at encoding, *F*(1, 49) = 5.36, *p* = .03, *η*_*p*_^2^ = .10, the advantage being for sober encoding (*M* = 3.50, *SE* = 0.08) over intoxicated encoding (*M* = 3.36, *SE* = 0.08).

**Table 2:** Summary of ANOVA results of likeness ratings as a function of alcohol conditions at encoding, construction, face composite condition and target face gender

	Likeness ratings		
Within subjects’ effects	*F*(1, 49)	*p*	*η*_*p*_^2^
Encoding	5.36	.025*	.10
Construction	0.01	.93	.00
Target gender	13.13	.001**	.21
Enc x Con	4.25	.05	.08
Enc x Gen	0.03	.87	.00
Con x Gen	6.63	.013*	.12
Between subject’s effects	*F*(2, 49)	p	*η*_*p*_^2^
Composite condition	8.27	.001**	.25
Enc x Comp	12.10	< .001***	.33
Con x Comp	1.88	.16	.07
Gen x Comp	8.13	.001**	.25
Enc x Con x Comp	5.62	.006**	.18
Enc x Con x Gen	9.77	.003**	.17
Enc x Con x Comp x Gen	6.59	.003**	.21

*Enc*, encoding; *Con*, construction, *Gen*, target gender; *Comp*, composite condition (full, external or internal features). *Significant *p* < .05; **significant *p* < .01; ***significant *p* < .001

**Fig. 2:**
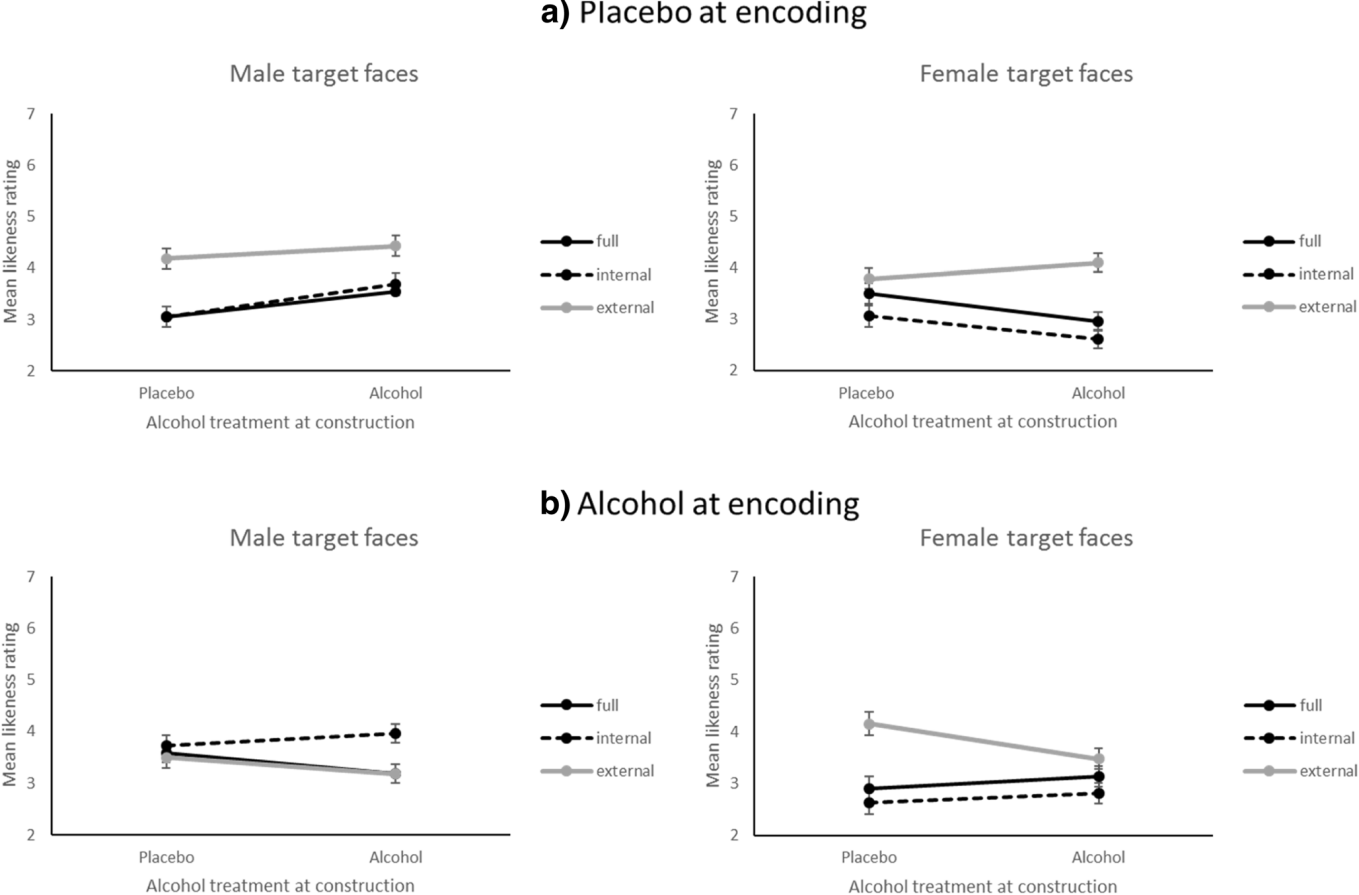
Mean likeness ratings by alcohol treatment at construction for male and female target faces. **a** Placebo at encoding. **b** Alcohol at encoding. Lines are added for interpretation only. Error bars represent ± 1 standard error of the mean; full, full facial composite (*n* = 17); internal, internal face features (*n* = 17); external, external face features (*n* = 18)

The final prediction related to the effect of alcohol intoxication at the time of face encoding on the external features of the composite. We predicted an advantage for external features only when these were salient (in our stimuli, the female hairstyles were salient compared to male hairstyles). This potential interaction between alcohol treatments, target face gender and composite condition is outlined below. Firstly, there was a significant main effect of target face gender, whereby the likeness of male target faces (*M* = 3.59, *SE* = 0.09) was higher than those of female faces (*M* = 3.26, *SE* = 0.09), *F*(1, 49) = 13.13, *p* = .001, *η*_*p*_^2^ = .21. As Table 2 shows, there were several significant interaction effects. The key interaction of interest for our prediction is the four-way interaction, which was significant, *F*(2, 49) = 6.59, *p* = .003, *η*_*p*_^2^ = 0.21, and this was followed up using simple main effects analyses which are corrected using the Sidak method in SPSS (Sidak provides tighter bounds than Bonferroni in the pairwise multiple comparison tests). In order to address our prediction relating to differentiated effects of alcohol treatments and face gender on likeness ratings for the composite stimuli, the likeness ratings for each combination of composite condition (full, internal and external) were compared at each combination of levels of the other three factors. These simple main effects are listed in Table 3, and visually represented in Fig. 2. The results show that, for male target faces, the advantage for external face features (external vs. internal: *p* = .001, external vs. full: *p* = .001) is eliminated when participants are intoxicated at the time of encoding (external vs. internal: *p* = .980, external vs. full: *p* = .735). These effects are illustrated in the left panels of Fig. 2 by comparing light grey lines between the top and lower graph. Furthermore, when participants are intoxicated at both encoding and construction, there is an advantage for internal male facial features, *p* = .009 (see Fig. 2, left panels, dashed lines). In contrast, for female target faces, the advantage for external face features is not affected by intoxication at the time of face encoding, but alcohol at encoding and construction eliminates the external feature advantage (external vs. full: *p* = .561, external vs. internal: *p* = .071). This is illustrated in the right panels of Fig. 2, by comparing the light grey lines. Notably, alcohol at either encoding or construction does not affect the likeness ratings for internal features of female target faces (dashed lines in right panel of Fig. 2).

**Table 3:** Simple main effects comparisons for full, internal and external facial composite stimuli, presented at each combination of levels of the other three factors (target face sex, treatment at encoding, treatment at construction)

Target face	Encoding	Construction	Comparison	Mean difference	SE	*p*	95% CI	Cohen’s *d*′
Male	Placebo	Placebo	Full-Int	0.00	0.28	1.00	[− 0.70, 0.70]	0.00
			Full-Ext	− 1.12	0.28	.001***	[− 1.81, − 0.43]	0.70
			Int-Ext	− 1.12	0.28	.001***	[− 1.81, − 0.43]	0.60
		Alcohol	Full-Int	− 0.15	0.30	.946	[− 0.88, 0.59]	0.08
			Full-Ext	− 0.89	0.29	.012*	[− 1.61, − 0.16]	0.54
			Int-Ext	− 0.74	0.29	.044*	[− 1.46, − 0.02]	0.40
	Alcohol	Placebo	Full-Int	− 0.15	0.26	.921	[− 0.78, 0.49]	0.11
			Full-Ext	0.09	0.25	.980	[− 0.54, 0.72]	0.06
			Int-Ext	0.24	0.25	.735	[− 0.39, 0.86]	0.14
		Alcohol	Full-Int	− 0.78	0.26	.011**	[− 1.42, − 0.15]	0.51
			Full-Ext	0.01	0.25	1.00	[− 0.61, 0.63]	0.01
			Int-Ext	0.79	0.25	.009**	[0.17, 1.41]	0.52
Female	Placebo	Placebo	Full-Int	0.44	0.29	.340	[− 0.27, 1.15]	0.25
			Full-Ext	− 0.29	0.28	.666	[− 0.99, 0.41]	0.16
			Int-Ext	− 0.73	0.28	.037*	[− 1.43, − 0.04]	0.50
		Alcohol	Full-Int	0.34	0.26	.478	[− 0.29, 0.97]	0.24
			Full-Ext	− 1.14	0.25	.000***	[− 1.77, − 0.51]	0.78
			Int-Ext	− 1.48	0.25	.000***	[− 2.11, − 0.85]	0.91
	Alcohol	Placebo	Full-Int	0.26	0.33	.808	[− 0.55, 1.07]	0.14
			Full-Ext	− 1.26	0.32	.001***	[− 2.05, − 0.46]	0.61
			Int-Ext	− 1.52	0.32	.000***	[− 2.32, − 0.72]	0.85
		Alcohol	Full-Int	0.32	0.29	.610	[− 0.39, 1.04]	0.22
			Full-Ext	− 0.34	0.29	.561	[− 1.05, 0.37]	0.21
			Int-Ext	− 0.66	0.29	.071	[− 1.37, 0.04]	0.35

Full, full face composite; Int, internal features of composite; Ext, external features of composite. *Significant *p* < .05; **significant *p* < .01; ***significant *p* < .001

## Discussion

We believe that this study is the first to investigate the effect of alcohol intoxication on the construction of facial composite images. While there has been a recent focus on examining the reliability of intoxicated witnesses performing line-up identifications, the analogous investigation for this forensically important alternative means of identifying potential perpetrators has not been studied. The experiment used a measure of likeness ratings to evaluate the quality of facial composite images constructed by participants who had been allocated to one of four factorial combinations of alcohol and placebo beverages at encoding and retrieval. The facial composite images were presented to the rating participants as either the full face or digitally edited stimuli showing only internal or external facial features. The aims were to examine the effects of alcohol on face encoding and face composite construction, to see if intoxication differentially impairs the quality of internal and external composite regions, and to see whether these effects vary between male and female target faces, as a function of hair distinctiveness.

Previous research on unfamiliar face learning has revealed clear processing advantages for external facial features in the context of Western, Caucasian faces and participants (Bruce et al. 1999; Hancock et al. 2000, Longmore et al. 2015; but for cultural differences see Megreya and Bindemann 2017). We therefore expected external facial features to be more accurately represented in facial composites than internal facial features, at least in those produced by participants who were sober during target face encoding and construction. The results confirmed this hypothesis as external features were rated as having a better overall likeness to the target face than the internal or whole face composites.

Based on the findings of Harvey (2014) and Dysart et al. (2002), which both suggest that alcohol at encoding restricts face encoding, we predicted that composites produced by participants intoxicated when viewing the target video would be of worse quality than those produced by participants who were sober at encoding. This prediction was also confirmed, with composites produced by participants intoxicated at encoding receiving lower likeness ratings than those constructed by their sober-encoding counterparts. However, Harvey (2014) and Dysart et al. (2002) offer different conclusions concerning the specific nature of restricted face encoding under alcohol. The former provides eye-tracking evidence of a narrowing of fixations to the centre of the face, which leaves face recognition performance unharmed, while the latter authors posit that intoxicated witnesses are likely to make more false identifications than sober controls because they are too narrowly focused on distinctive external aspects of target faces, such as hairstyles. Both studies make reference to AMT in their discussion of findings, but apply AMT to their findings slightly differently. Harvey (2014) refers to restricted processing under alcohol, which happens to narrow central fixations in the absence of salient external features, while Dysart et al. (2002) emphasise the lure of external face salience under restricted processing capacity.

The results of our study complement both explanations. The key finding was that alcohol at encoding eliminated the advantage for external face parts, but only for male faces. Alcohol at both encoding and retrieval furthermore resulted in an advantage for internal male face parts (see Fig. 2). In line with the suggestion of Dysart et al. (2002), alcohol therefore appears to have narrowed the attention of intoxicated viewers to the distinctive hairstyles of our female target faces, but onto the internal features of our male target faces, who all happened to wear short and somewhat uniform haircuts. This latter finding is in keeping with Harvey’s (2014) eye-tracking data, in which alcohol restricted encoding scanpaths to the central areas of the target faces, all of which were also male with short and unremarkable hairstyles. We therefore propose that in the absence of distinctive external features (such as long, styled hair), alcohol intoxication leads to a narrowing of attention on to internal features of unfamiliar faces during face learning.

The final key finding of the present study was that the advantage for higher quality external composite features was eliminated for composites produced by participants intoxicated at both encoding and retrieval. To date, only Dysart et al. (2002) and Hilliar et al. (2010) have reported face memory impairments by participants intoxicated at both encoding and retrieval. Dysart et al. (2002) asked participants to identify (from a single photograph) a person with whom they had interacted moments earlier, and found a positive association between level of intoxication and the likelihood of making a false identification. While Hilliar et al. (2010) found that participants intoxicated during the encoding and retrieval phase of an old-new face recognition task made fewer hits and more false alarms for “other race” faces than sober controls. The present study, along with that of Dysart et al. and Hilliar et al., suggests that alcohol intoxication at encoding and retrieval impairs face processing—at least in face recognition, showup identification and composite construction tasks.

Curiously, alcohol at encoding and retrieval has not been found to impair face identification in line-up studies (e.g. Altman et al. 2018) though we believe this discrepancy may be explained by the fact that, in line-up studies, the target face must typically be encoded within a complex stimulus scene (such as a staged crime) amongst other competing stimuli (such as the location of the staged crime, other scene actors, etc.). When the target face is one of a number of faces presented in a scene, participants are more likely to process it holistically, paying relatively little attention to specific facial features. In the present study and that of Dysart et al. (2002) and Hilliar et al. (2010), however, each target face was presented alone and thus with minimal distraction from competing visual stimuli. In these examples, when the target face is the only visual stimulus that participants are required to encode, they are more likely to analyse constituent face parts, with some features (e.g. eyes, nose and hair) inevitably attracting more attention than others. For our sober encoders, the external region of the target face was likely the most salient aspect of the stimulus as each of the faces presented in the video clips were unfamiliar to them (e.g. Longmore et al. 2015). However, Harvey’s (2014) finding of a significant narrowing of visual scanpaths for intoxicated participants on to the nose region of unfamiliar male targets indicates differential face processing under alcohol, and this restricted scanning may explain why composites constructed by participants intoxicated at encoding in our study were generally worse than those produced by sober encoders. We also suggest that the re-administration of alcohol at retrieval worsened the quality of external composite regions in particular because intoxication impairs the resource intensive recall process required to construct the composites (Schreiber Compo et al. 2017; Söderlund et al. 2005), with memory for external face regions more likely to have been impaired by alcohol at encoding. We aim to evaluate these suggestions in future studies with the application of eye tracking to the present design.

While we demonstrate that low to moderate levels of alcohol intoxication at encoding have a small but significant detrimental effect on the quality of composite images, our effects relating to target face gender are confounded with the hairstyles of target identities. As it is impossible to determine whether the present alcohol effects are caused by salient hairstyles, female faces, or a combination of both, our conclusion that salient hairstyles drives the alcohol effect remains speculative until the effects of hairstyles and target face gender are deconfounded. A further limitation of our study is that the intoxication levels of real witnesses can be far higher than the levels elicited here, and recent work indicates that BACs greater than 0.1% have a more pronounced impact on memory performance (e.g. Altman et al. 2018; Crossland et al. 2016). We therefore expect face composite quality to be significantly more compromised at high intoxication levels and recommend this prediction be tested in the field where heavy alcohol consumption is commonplace. A further way to strengthen the ecological validity of our study is to incorporate a face composite naming measure. In forensic contexts, composite images are used to identify perpetrators and some members of the public who recognise them (from a composite) may also be personally familiar with them. It is therefore important to conduct a naming study (Frowd et al. 2007) to evaluate the quality of facial composites produced by intoxicated witnesses, to support the likeness rating approach adopted here.

In conclusion, composite images constructed by witnesses who were moderately intoxicated when they encoded the target face are generally judged to be of lesser quality than those constructed by sober witnesses. More specifically, intoxication during face learning impairs the encoding of non-distinct external features critical for the discrimination of unfamiliar faces, and this effect is exacerbated in witnesses that remain intoxicated during face retrieval and composite construction. Further investigation into the mechanisms by which intoxication may impact face processing is therefore warranted.

## Footnotes

1 While it could be reasoned that alcohol at both encoding and retrieval should facilitate composite construction via state-dependent learning effects (Tulving and Thompson 1973), there is currently no strong evidence to support this view in the literature (see Schreiber Compo et al. (2017) for a review). We assume this is because alcohol restricts encoding leaving fewer traces of the stimulus event. The reinstatement of intoxication at test may provide additional cues for retrieval but it will not enrich the impoverished trace.
